# DOT1L promotes immune evasion in lung adenocarcinoma through H3K79me2-mediated epigenetic activation of immune checkpoints

**DOI:** 10.3389/fimmu.2026.1719299

**Published:** 2026-02-26

**Authors:** Xiwu Rao, Xiangjun Qi, Zhiqiang Chen, Jingrui Wang, Xiaoshu Chai, Lizhu Lin

**Affiliations:** 1 Guangzhou University of Chinese Medicine, Guangzhou, Guangdong, China; 2 Guangdong Provincial Hospital of Chinese Medicine, The Second Affiliated Hospital of Guangzhou University of Chinese Medicine, Guangdong Provincial Academy of Chinese Medical Sciences, Guangzhou, Guangdong, China; 3 Oncology Center, The First Affiliated Hospital of Guangzhou University of Chinese Medicine, Guangzhou, Guangdong, China; 4 Lingnan Medical Research Center, Guangzhou University of Chinese Medicine, Guangzhou, Guangdong, China

**Keywords:** DOT1l, H3K79 methylation, immune evasion, lung adenocarcinoma, STAT3/PD-L1 axis

## Abstract

The histone methyltransferase DOT1L, the sole enzyme catalyzing H3K79 methylation, is increasingly implicated in cancer progression, yet its role in shaping the tumor immune microenvironment (TME) remains unclear. Here, we demonstrate that DOT1L orchestrates immune evasion in lung adenocarcinoma (LUAD) through epigenetic activation of multiple immune checkpoints. Integrative analysis of TCGA and single-cell RNA-seq data revealed that high DOT1L expression correlates with poor prognosis, diminished cytotoxic immune-cell infiltration, and upregulation of inhibitory checkpoints (PD-L1, PD-1, LAG3, CD276, etc.). Mechanistically, ChIP-seq identified DOT1L-mediated H3K79me2 enrichment at promoters of JAK1/STAT3 genes, and some immune checkpoints, including LAG3, CD276, etc. Pharmacological DOT1L inhibition (SGC0946) suppressed the JAK1/STAT3/PD-L1 axis, reduced PD-1+ T cells in a vitro immune microenvironment. *In vivo*, SGC0946 attenuated lung metastasis, improved survival, and remodeled the TME by downregulating PD-L1, LAG3, and CD276 expression, reduced PD-1+ T cells subsets, and alongside with enhanced TNF-α, IFN-γ production. Clinical LUAD specimens further validated the correlation between DOT1L expression, STAT3 activation, and checkpoint upregulation, particularly in metastatic disease. Our findings identify DOT1L as an epigenetic linchpin of immune suppression.

## 1 Background

The histone methyltransferase Disruptor of Telomeric Silencing 1-Like (DOT1L) is the sole enzyme responsible for catalyzing methylation at histone H3 lysine 79 (H3K79) (1), a conserved epigenetic mark linked to transcriptional activation and elongation. Initially identified in yeast for its role in telomeric silencing, DOT1L has emerged as a critical regulator in mammalian cells, governing diverse biological processes, including cell cycle progression (2), and DNA damage repair (1). Notably, aberrant DOT1L activity has been implicated in oncogenesis, particularly in leukemias driven by MLL rearrangement (3), where DOT1L-mediated Histone H3 lysine 79 dimethylation (H3K79me2) facilitates the expression of pro-leukemic genes (4). Beyond hematological malignancies, growing evidence suggests that DOT1L expression is increased in various malignant tumor tissues, resulting in enhanced H3K79 methylation, and is closely related to poor prognosis (5–8). In non-small cell lung cancer (NSCLC), DOT1L’s oncogenic potential is increasingly recognized. Research found mutations in the catalytic domain of DOT1L promote lung cancer malignant phenotypes, but the mutation rate of DOT1L in lung cancer is only about 3% (9).

Tumor immune evasion is a critical hallmark of cancer progression, enabling malignant cells to escape surveillance and destruction by the immune system (10). Among immune cell populations, T cells, particularly CD8+ cytotoxic T lymphocyte, play a central role in anti-tumor immunity (11). However, tumors exploit multiple mechanisms to suppress T-cell function, creating an immunosuppressive microenvironment. The Programmed Cell Death Protein 1/Programmed Cell Death-Ligand 1(PD-1/PD-L1) axis is a well-characterized pathway where tumor-associated PD-L1 binds PD-1 on T cells, delivering inhibitory signals that dampen T-cell activation, proliferation, and effector functions (12). Similarly, Lymphocyte-Activation Gene 3(LAG3), expressed on exhausted T cells, interacts with MHC class II molecules on antigen-presenting cells or tumor cells, further suppressing T-cell responses (13). Another emerging checkpoint, Cluster of Differentiation 276(CD276, B7-H3), is overexpressed in many cancers and contributes to immune evasion by inhibiting T-cell infiltration and promoting regulatory T cell activity (14). The accumulation of dysfunctional or “exhausted” T cells, characterized by sustained checkpoint expression, further compromises anti-tumor immunity. Targeting these pathways with immune checkpoint inhibitors (ICIs), such as anti-PD-1/PD-L1 or anti-LAG3 therapies (15), has revolutionized cancer treatment.

Here, we investigate DOT1L’s role in shaping the immunosuppressive landscape of Lung Adenocarcinoma (LUAD) by integrating bioinformatics, epigenomics, and animal models, clinical samples, we provide a mechanistic rationale for targeting DOT1L to remodel the tumor immune microenvironment. Mechanistically, DOT1L-mediated H3K79me2 modification concurrently upregulates multiple immune checkpoints, which creating a synergistic immunosuppressive network that inhibits T cell infiltration and function. Animal models show that pharmacological inhibition of DOT1L significantly reduces the expression of these immune checkpoints, reverses T cell exhaustion, and exhibited the anti-tumor efficacy *in vivo*. Further investigations are warranted to elucidate the detailed molecular mechanisms.

## 2 Methods

### 2.1 Data analysis and immune microenvironment stratification using The Cancer Genome Atlas database

Transcriptomic profiles and corresponding clinical information for tumor were obtained from TCGA database. Differential expression analysis of DOT1L between tumor and normal tissues was performed across multiple cancer types to assess its pan-cancer expression pattern, and the expression of DOT1L in lung cancer samples with different TNM stages. Immune cell infiltration was quantified in TCGA-LUAD tumor samples using single-sample gene set enrichment analysis (ssGSEA), implemented in the IOBR package (v0.99.8), based on gene signatures for 28 immune cell subsets curated from MSigDB. Patients were stratified into high, medium, and low DOT1L expression groups according to tertiles, yielding n = 171/171/171 samples per group. Differences in ssGSEA scores across immune cell subsets, as well as the expression of immune-related genes (including ligands, receptors, and co-stimulatory/co-inhibitory molecules), were assessed using two-sided Kruskal - Wallis tests. P values were adjusted for multiple comparisons using the Benjamini - Hochberg false discovery rate (BH-FDR) procedure.

### 2.2 Single-cell landscape analysis using ScCancerExplorer platform

Single-cell expression data for LUAD were obtained from the ScCancerExplorer platform (https://bianlab.cn/scCancerExplorer/explore/search), based on the study by Luo et al (16). We visualized the single-cell atlas of LUAD to examine the distribution of DOT1L expression across various cell types, including lymphatic cells, myeloid cells, and fibroblasts.

### 2.3 Kaplan–Meier survival analysis

Kaplan–Meier survival curves were generated using the KM Plotter online tool (https://kmplot.com/analysis/). LUAD patients (n=2,166) were stratified by DOT1L expression levels, and overall survival (OS) was compared. Hazard ratios (HRs) and 95% confidence intervals (CIs) were calculated to assess prognostic significance.

### 2.4 Cell culture and treatments

Human lung adenocarcinoma cell lines (A549 and H1975, PC9) were maintained in RPMI-1640 medium supplemented with 10% fetal bovine serum (FBS) at 37°C in 5% CO2. For DOT1L inhibition experiments, cells were treated with the selective DOT1L inhibitor SGC0946 (Aladdin, 1561178-17-3) at concentrations ranging from 0.5 to 2nM for 48 hours. To rescue STAT3 activity, cells were co-treated with 2 nM STAT3 agonist ML115 (MedChemExpress, HY-111152) for 48 hours. Control groups received equivalent volumes of DMSO vehicle.

### 2.5 Chromatin immunoprecipitation sequencing and data analysis

A549 Cells were fixed with formaldehyde to crosslink DNA-protein complexes, followed by lysis to extract chromatin. Chromatin was sheared into 200–500 bp fragments using sonication. Target protein-DNA complexes were enriched using H3K79me2 specific antibody(Active Motif, 39143,5μl each), followed by magnetic bead-based purification. Immunoprecipitated DNA was reverse-crosslinked, purified, and subjected to end repair, A-tailing, and adapter ligation. Libraries were amplified by PCR and sequenced on an Illumina platform. Raw sequencing reads (FASTQ files) were trimmed using Trimmomatic to remove adapters and low-quality bases (Q-score < 20). FastQC (v0.11.9) was employed to assess data quality, including: Base quality distribution (Q20 ≥ 90% expected). Sequence error rate (typically higher at read ends). Nucleotide composition (A/T and C/G balance). GC content (checked for contamination if multi-modal). Clean reads were aligned to the reference genome using Bowtie2 (v2.4.4) with default parameters. Mapped reads were filtered to remove PCR duplicates, low-quality alignments, and organelle-derived sequences. Genome-wide profiles and locus-specific signals were visualized using IGV Browser. All analyses were conducted in R (v4.2.0) or Python (v3.9), with significance thresholds adjusted for multiple testing (Benjamini-Hochberg FDR < 0.05).

### 2.6 Co-culture system with PBMCs

Peripheral blood mononuclear cells (PBMCs) were obtained from the American Type Culture Collection (ATCC, Manassas, VA, USA). For tumor-immune interaction studies, co-culture was conducted using transwell chambers (0.4-μm pore, Corning Incorporated, NY, USA) in 12-well plates to prevent cell migration between chambers. Initially, A549 (2×10^5^ cells/well) were incubated in the transwell upper chambers at 37°C with 5% CO_2_ for 6 h to allow adhesion. Subsequently, PBMCs (2×10^6^ cells/well) in 500 μL of complete RPMI-1640 medium in the presence or absence of SGC0946. The co-culture system was maintained at 37°C in 5% CO_2_ for 48h.

### 2.7 Western blot analysis

Cell protein lysates were extracted using RIPA buffer supplemented with protease and phosphatase inhibitors. Equal amounts of protein (30 μg per lane) were separated by 10% SDS-PAGE, transferred to PVDF membranes, and probed with the following primary antibodies: Anti-DOT1L (1:1000, Cell Signaling Technology, USA), Anti-H3K79me2 (1:1000, Cell Signaling Technology, USA), Anti-JAK1, Anti-STAT3, Anti- STAT3 phosphorylation at Tyr705 (p-STAT3), Anti-PD-L1, Anti-GAPDH (1:1000, Proteintech, USA). Blots were developed using HRP-conjugated secondary antibodies and visualized via chemiluminescence (ECL, Bio-Rad, USA).

### 2.8 Quantitative PCR analysis

For the synthesis of cDNA, total RNA underwent reverse transcription utilizing the PrimeScript RT Reagent Kit (Vazyme, Nanjing, China). The following primers were listed in **Table 1**. Relative mRNA expression levels were calculated via the 2−ΔΔCt method, normalized to GAPDH.

**Table 1:** Primers.

Genes	Primers (5’- 3’)
STAT1	F: ATGGCAGTCTGGCGGCTGAATT
	R: CCAAACCAGGCTGGCACAATTG
STAT3	F: ATGGCCCAATGGAATCAGC
	R: TCACATGGGGGAGGTAGCGC
JAK1	F: ACCGAGGACGGAGGAAAC
	R: ACTGCCGAGAACCCAAAT
RELA(p65)	F: CCCACGAGCTTGTAGGAAAGG
	R: GGATTCCCAGGTTCTGGAAAC
GAPDH	F: TGTTGCCATCAATGACCCCTT
	R: CTCCACGACGTACTCAGCG

### 2.9 *In vivo* mouse xenograft tumorigenesis experiments

A total of 100 male C57BL/6 mice (6–8 weeks old) were used in this study. The Lewis lung carcinoma (LLC) cell line stably expressing luciferase (LLC-luc) was cultured in DMEM supplemented with 10% FBS and 1% penicillin/streptomycin. For the pulmonary metastasis model, LLC-luc cells (1×10^6 cells in 100 μL PBS) were injected into the tail vein of each mouse. Tumor engraftment was confirmed by bioluminescence imaging (BLI) using an IVIS Spectrum imaging system (PerkinElmer). Randomization/Blinding for Animal: After tumor engraftment confirmation, mice were randomly assigned to four groups using a random number table. Exclusion Criteria: Mice were excluded if they failed tumor engraftment (no bioluminescence signal).Mice were randomized into four groups (n = 12/group):①Control group (Normal Saline, intraperitoneal injection(i.p), once daily), ② PD-1 inhibitor group (BioXCell, USA, 200 μg i.p, twice weekly), ③ SGC0946-low does group (20 mg/kg, i.p, once daily), and ④SGC0946-high does group (40 mg/kg, i.p, once daily). Treatment was initiated 24h after randomization and continued for 3 weeks. Body weight was monitored every week. At the end of the experiment, the mice tumor engraftment was confirmed by BLI, and followed by euthanized. Mice were initially placed in an induction chamber and exposed to 4% isoflurane mixed with pure oxygen (flow rate: 1 L/min), with body temperature maintained at 37°C using a heating pad throughout the procedure. At the experimental endpoint, euthanasia was performed by cervical dislocation under maintained anesthesia. Organ collection was performed immediately post-euthanasia: weighed and stored at 80°C or fixed in formalin for subsequent determination of tumor tissue protein expression or immunohistochemistry(IHC). These experiments were approved by the Animal Experimentation Ethics Committee of Guangzhou University of Chinese Medicine (Ethical approval number, 20240823004).

### 2.10 Flow cytometry assay

Mouse lung tissue was cut into small pieces and digested in DMEM basic medium with 1 mg/mL type IV collagenase and 200 μg/ml deoxyribonuclease I at 37°C for 1 h. Cells were counted and dispersed in PBS at a concentration of 1 ×10^6^ cells/ml. Then, the cells were stained with 1 μl of fluorescently conjugated antibody (Biolegend, USA) in 100 μl of PBS at 4°C for 30 min. The specific antibodies include: anti-PD-1-PerCP-eFlour710, anti-CD45-eFlour506, anti-CD3-AF488, anti-CD4-APC/Fire810 and anti-CD8-BV570. The samples were analyzed via flow cytometry using a Cytek Aurora flow cytometer, facilitating the comprehensive identification and quantification of various immune cell types. The *in vitro* experimental section was performed using BD flow cytometry-specific antibodies (BD Biosciences, USA), including anti-CD45- BV510, anti-CD3-FITC, anti-CD4-APC, anti-CD8-PerCP-CY5.5, and anti-PD1-BV605. After processing and staining, the samples were comprehensively analyzed using flow cytometry.

### 2.11 ELISA assay

Freshly harvested mouse lung tumor tissues were weighed and homogenized in ice-cold PBS (containing 1x protease inhibitor cocktail (Beyotime, China) at a ratio of 100 mg tissue per 1 mL buffer. The homogenates were centrifuged at 12,000 x g for 15 min at 4°C, and the supernatants were collected for ELISA analysis. Protein concentrations were determined using a BCA Protein Assay Kit (Beyotime, China) to normalize cytokine levels. The concentrations of Tumor Necrosis Factor-alpha (TNFα), Interferon-gamma(IFNγ) and Granzyme B(GzmB) in the supernatants were determined with a Mouse TNFα, IFNγ, GzmB ELISA kit (Beyotime, China) according to the manufacturer’s instructions.

### 2.12 Patients and clinical samples

A total of 40 lung cancer tissues and their corresponding adjacent normal tissues were obtained from patients diagnosed with LUAD at the First Affiliated Hospital of Guangzhou University of Chinese Medicine. The conduct of this study was authorized and approved by the Human Research Ethics Committee of the First Affiliated Hospital of Guangzhou University of Chinese Medicine(Ethical approval number, K-2024-035).

## 3 Results

### 3.1 Clinical relevance of DOT1L in NSCLC based on TCGA analysis

**Figure 1:**
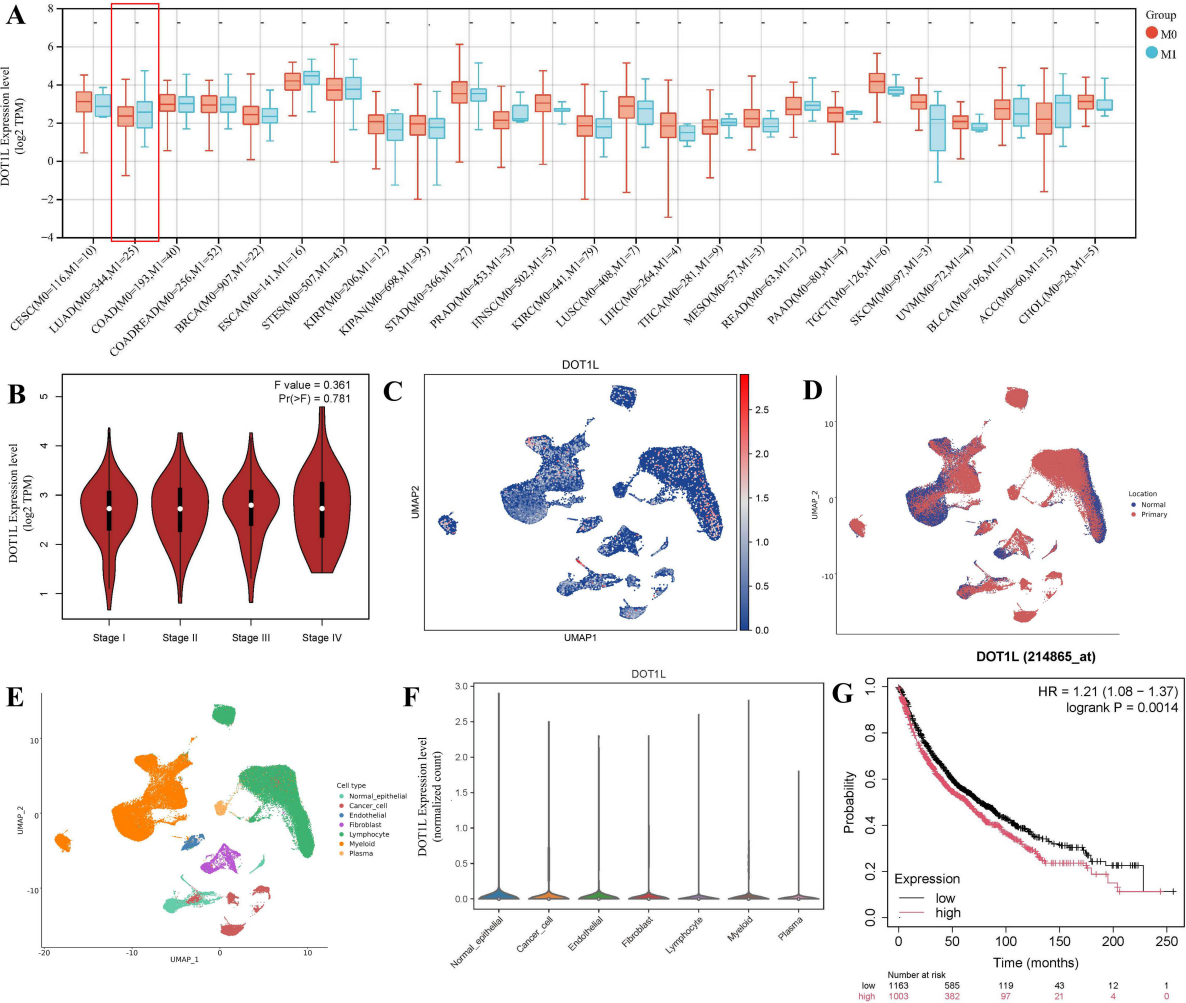
DOT1L expression in LUAD and its association with clinical outcomes. **(A)** DOT1L expression in TCGA LUAD samples with distant metastasis versus non-metastatic samples. **(B)** DOT1L expression levels in TCGA LUAD samples with stage I, II, III, IV respectively. **(C)** Single-cell expression profiling of DOT1L in LUAD tissues using integrated scRNA-seq data. **(D)** Single-cell expression profiling exhibits DOT1L expression in primary tumor tissues and normal lung tissues. **(E)** Single-cell expression profiling exhibits DOT1L expression across different cell types. **(F)** Expression levels of DOT1L in different cell types. **(G)** Kaplan-Meier survival analysis of DOT1L expression in LUAD.

Transcriptomic data from TCGA were used to investigate the differential expression of DOT1L between tumor and normal tissues across multiple cancer types. Pan-cancer analysis revealed that DOT1L expression varied significantly among different cancers. We analyzed DOT1L expression in LUAD samples with distant metastasis (n = 344) versus those without distant metastasis (n = 25), and found DOT1L expression was higher in metastatic samples, but the difference did not reach statistical significance (**Figures 1A, B**). Next, we utilized the ScCancerExplorer platform to examine the single-cell expression profile of DOT1L in LUAD. This analysis includes 35 NSCLC tissues and 18 normal lung tissues. We visualized the single-cell atlas of LUAD and the distribution of DOT1L expression across different cell types. Higher expression of DOT1L was observed in primary tumor than normal lung tissues (**Figures 1C, D**). Besides, DOT1L expression was observed higher in cancer cells than endothelial cells (**Figures 1E, F**). Kaplan-Meier survival analysis was conducted using KM Plotter (https://kmplot.com/analysis/), showing that high DOT1L expression was significantly associated with poorer overall survival in 2,166 LUAD patients (HR = 1.21; 95% CI: 1.08–1.37, *P* < 0.05) (**Figure 1G**). These findings suggest that DOT1L expression tends to be positively correlated with tumor progression. But DOT1L showed no significant correlation with tumor stage, differentiation grade, or other clinicopathological features (**Supplementary Figures S1A-E**), so we further investigated its potential role in shaping the tumor immune microenvironment to explore alternative mechanisms underlying its possible involvement in cancer progression.

### 3.2 Association between DOT1L expression and immunosuppressive tumor microenvironment in LUAD

**Figure 2:**
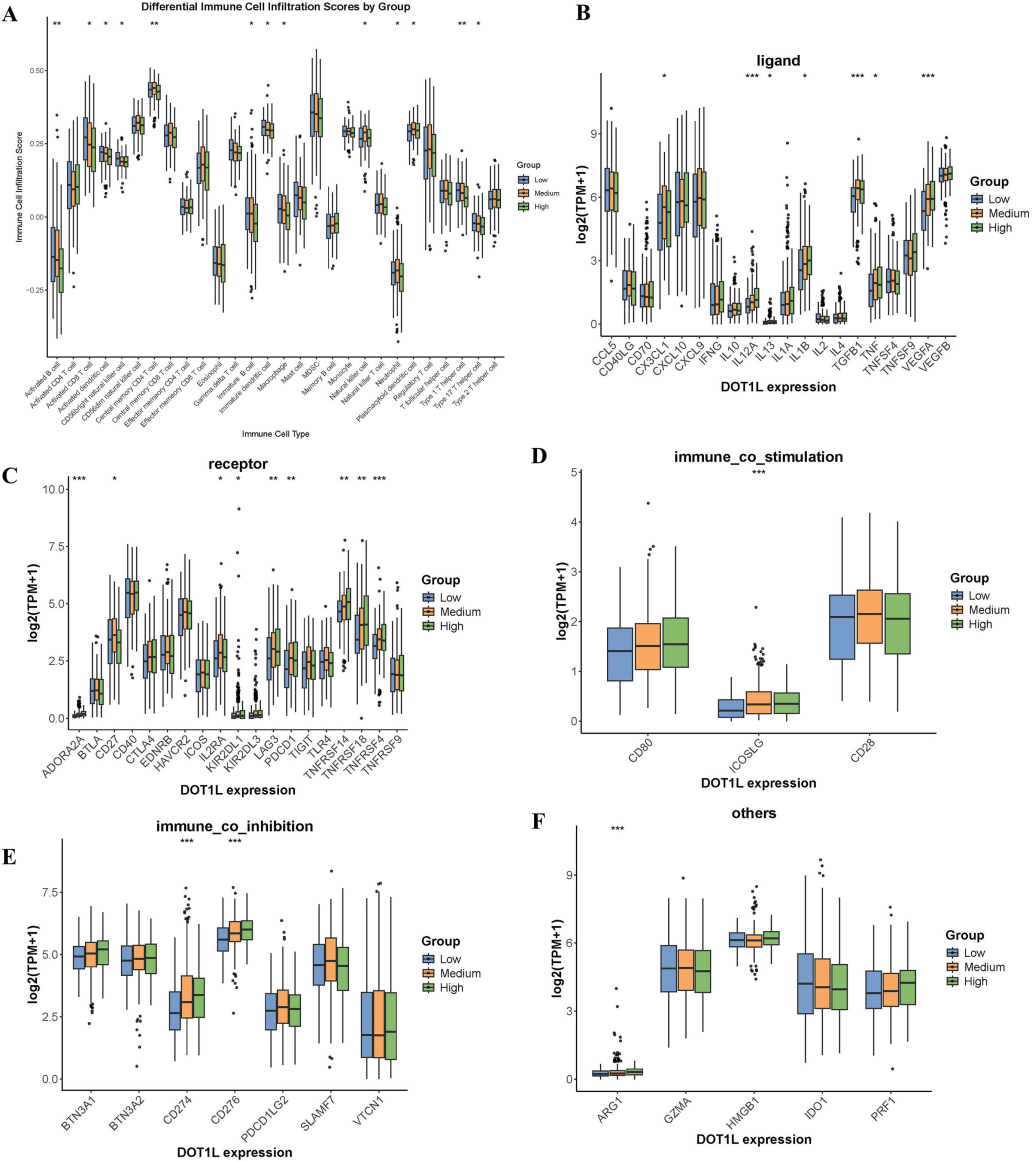
Evaluation of DOT1L Expression with Immune Cell Infiltration and Immune Checkpoint in LUAD by ssGSEA. **(A)** Correlation between DOT1L expression and immune cell infiltration in the TCGA-LUAD cohort. **(B-E)** Correlation between DOT1L expression and immune checkpoint-related genes, including **(B)** Ligands, **(C)** Receptors, **(D)** Immune co-stimulatory molecules, and **(E)** Immune co-inhibition molecules. **(F)** Other immune checkpoint-related genes (*P < 0.05, **P < 0.01, ***P < 0.001).

We explored the relationship between DOT1L expression and the tumor immune microenvironment (TME) in the TCGA-LUAD cohort. LUAD samples were stratified into high, medium, and low expression groups based on DOT1L levels. Immune cell infiltration scores were calculated using the ssGSEA algorithm. After analysis, DOT1L expression exhibited a negative correlation with the infiltration of various immune cell populations, including activated B cells, activated CD8^+^ T cells, T helper 1 (Th1) cells, and T helper 17 (Th17) cells (**Figure 2A**). Activated CD8^+^ T cells are the primary executors of tumor cell killing through granzyme/perforin-mediated cytotoxicity and Interferon-gamma (IFN-γ) production (17). Their infiltration is generally associated with better prognosis (18). Activated CD4^+^ T cells serve as central coordinators of antitumor immunity by supporting CD8^+^ T cell function, promoting dendritic cell (DC) maturation, and secreting cytokines that modulate immune responses (19). CD4^+^ T cells can also differentiate into helper T cells (20), such as Th1 cells, a subset of CD4^+^ T helper cells that are characterized by their production of IFN-γ, Tumor Necrosis Factor-alpha (TNF-α), and IL-2 (21), and Th1 responses are generally associated with antitumor immunity due to their ability to activate CD8^+^ T cells (22), enhancing tumor cell killing (23). This is consistent with the notion that DOT1L-mediated epigenetic regulation may contribute to an immunosuppressive TME. 

Besides, persistent antigen exposure leads to T-cell exhaustion, characterized by upregulated inhibitory immune checkpoint molecules (e.g., PD-1, LAG3, etc) (24–26). Notably, DOT1L exhibits a strong positive correlation with key immune checkpoint molecules, including several ligands such as CX3CL1, IL12A, IL1B, TGFB1, TNF, and VEGFA (**Figure 2B**); Receptor genes such as ADORA2A, KIR2DL1, LAG3, PDCD1, TNFRSF14, TNFRSF18, and TNFRSF4 (**Figure 2C**); Co-stimulatory gene ICOSLG (**Figure 2D**); Co-inhibitory molecules including CD274 (PD-L1), and CD276 (B7-H3) (**Figure 2E**), some other genes, including ARG1 (**Figure 2F**), and antigen presentation genes HLA-DRA, MICA, MICB (**Supplementary Figure S2**).

These findings suggest that DOT1L may facilitate immune escape by upregulating inhibitory checkpoints and dampening cytotoxic immune cell infiltration. So we further investigated the regulatory targets and mechanistic roles of DOT1L in LUAD to elucidate its impact on immune evasion and tumor progression.

### 3.3 ChIP-seq identifies DOT1L-mediated H3K79me2 as an epigenetic activator of immune evasion genes

**Figure 3:**
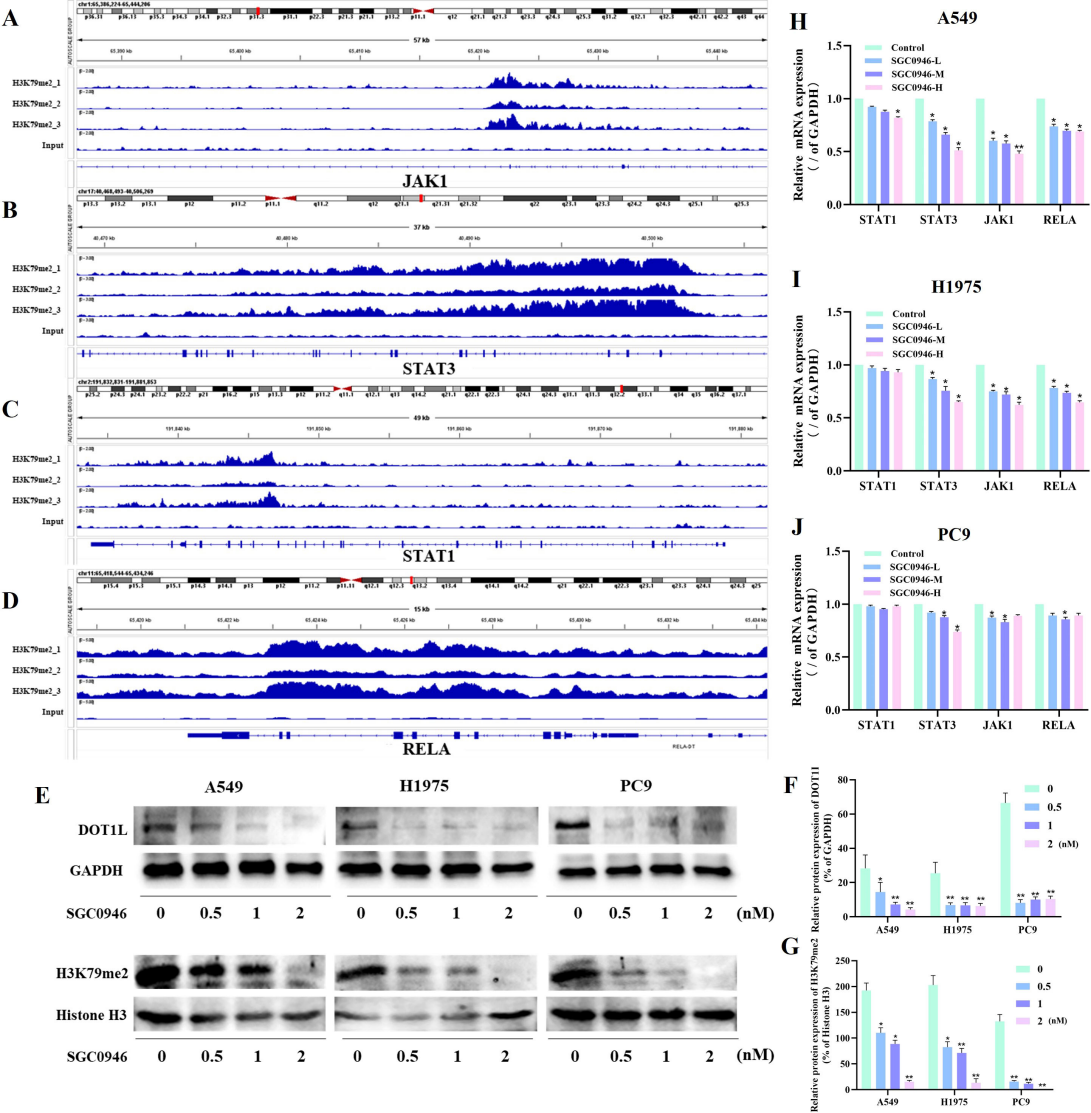
DOT1L-mediated H3K79me2 regulates immune evasion-related gene expression. **(A-D)** ChIP-seq profiles showing H3K79me2 enrichment in the gene regions of JAK1, STAT3, STAT1, and RELA in A549 cells. **(E-G)** Western blot analysis detected DOT1L and H3K79me2 protein levels after treatment with the DOT1L inhibitor SGC0946. GAPDH served as a loading control. **(H-J)** qPCR validation of STAT3, JAK1, and RELA mRNA downregulation upon SGC0946 treatment in three independent cell lines (*P < 0.05, **P < 0.01 versus control.).

DOT1L is the exclusive histone methyltransferase capable of catalyzing methylation at histone H3 lysine 79 (H3K79) (27), a critical regulator of transcriptional activation. Among the methylation states (mono-, di-, and tri-methylation), H3K79 dimethylation (H3K79me2) is strongly associated with gene activation transcription (28). We performed ChIP-seq for H3K79me2 and found that a cohort of immune evasion-related genes promoter regions were enriched. Some candidate genes were identified in the above prior bioinformatics analysis, including LAG3, CD276, TGFB1, HLA-DMA, and MICA (**Supplementary Table S1**). KEGG pathway enrichment analysis further revealed these genes with critical immune regulatory pathways, such as PD-L1/PD-1 checkpoint signaling, T cell receptor activation, Toll-like receptor signaling, Interleukin(IL)-17 signaling, Th17 differentiation and Th1/Th2 differentiation (**Supplementary Table S2**). Among the genes enriched in these pathways, we identified several key signaling molecules (NFKB1, MAPK1, PIK3CB, STAT1, STAT3, TRAF6, JAK1, RELA, FOS, and JUN), and found JAK1, STAT1, STAT3, RELA showed significant H3K79me2 modifications in their promoter regions (**Figures 3A-D**). Notably, the H3K79me2 signals were particularly strong in the promoter regions of JAK1, STAT3, and RELA, suggesting these genes may be preferentially regulated by DOT1L-mediated histone modification.

To validate these findings, we attempted to generate DOT1L-overexpressing and knockdown cell lines using a lentiviral system. However, due to the large size of the DOT1L gene fragment, stable transduction was challenging. As an alternative approach, we treated cells with DOT1L inhibitors (SGC0946). We first performed dose-dependent CCK-8 viability assays across multiple LUAD cell lines using SGC0946 at concentrations ranging from 0.5 nM to 5 nM for 48 hours. The results demonstrated that SGC0946 at concentrations up to 2 nM did not induce significant cytotoxicity, with cell viability remaining above 90% (**Supplementary Figure S8A**). Additionally, dose-dependent CCK-8 viability assays were conducted on PBMCs. Treatment of PBMCs with SGC0946 across the same concentration range (0.5nM to 5nM) for 48 hours showed no significant reduction in cell viability at concentrations 0.5–2 nM (**Supplementary Figure S8B**). Based on these findings, we selected concentrations of 0.5 nM, 1 nM, and 2 nM as the low-, medium-, and high-dose gradients of SGC0946 for subsequent *in vitro* experiments.

Western Blotting confirmed protein reduction in DOT1L and H3K79me2 levels after treatment of SGC0946 (**Figures 3E-G**). Additionally, qPCR analyses in three independent cell lines demonstrated significant downregulation of STAT3, JAK1, RELA at mRNA levels (**Figures 3H-J**). Besides, we also focused on the ChIp-seq signalings in the gene promoter regions of LAG3, CD276, TGFβ1, CD274, and found LAG3, CD276 showed significant H3K79me2 modifications in their promoter regions (**Supplementary Figure S3**). These results suggested that DOT1L-mediated H3K79me2 may epigenetically modulates immune evasion-related genes, potentially contributing to tumor immune escape. To demonstrate H3K79me2 is functionally required for immune-related genes transcription, we treated H1975 cells with SGC0946 for 48 hours and performed ChIP-qPCR to assess H3K79me2 enrichment at the promoter regions of JAK1, STAT3 and CD276. The results showed that concentrations of 1–2 nM effectively inhibited H3K79me2 deposition at the promoters of JAK1, STAT3 and CD276 (**Supplementary Figure S10**). Further functional studies are warranted to elucidate the function of DOT1L activity in lung cancer immune regulation.

### 3.4 The JAK1/STAT3/PD-L1 signaling axis represents a core molecular mechanism underlying DOT1L-induced immune escape

Among the multiple immune checkpoints, PD-L1 reigns supreme as the core molecular of immune evasion (29). Based on the above analyses, DOT1L-mediated H3K79me2 epigenetically activates JAK1, STAT3, which are enriched in the PD-L1/PD-1 checkpoint pathway (30), and ssGSEA bioinformatics also revealed that PD-L1 is positively related with DOT1L expression. This suggests that DOT1L may indirectly modulate PD-L1 expression through upstream signaling pathway. To test this hypothesis, we further investigated the effect of DOT1L inhibition on PD-L1 expression and its underlying molecular mechanisms.

**Figure 4:**
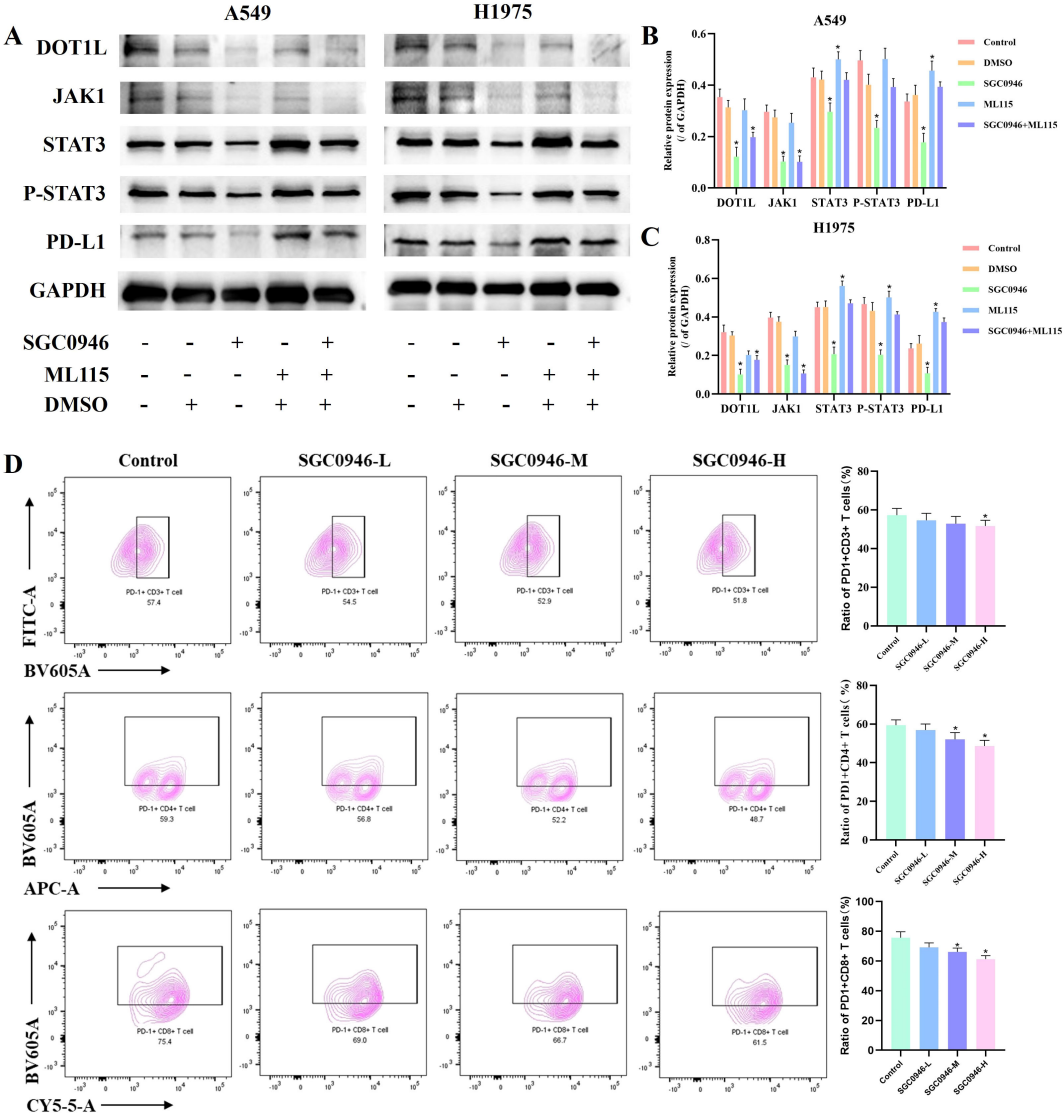
DOT1L promotes immune evasion in LUAD by activating the JAK1/STAT3/PD-L1 axis and inducing T-cell exhaustion. **(A-C)** Western blot analysis detected protein expression of DOT1L, JAK1, STAT3, p-STAT3 and PD-L1 in A549 and H1975 cells. **(D)** Flow cytometry analysis of PD-1+ T cell subsets in the co-cultured system of PBMCs with A549 cells(*P < 0.05 versus control).

We hypothesized that DOT1L epigenetically potentiating the JAK/STAT3 signaling axis. Activated STAT3 phosphorylation at Tyr705(p-STAT3) may directly bind to conserved response elements in the PD-L1 promoter, thereby enhancing its transcription (31). Western blot validation showed that DOT1L inhibition suppressed JAK1, STAT3, p-STAT3, PD-L1 protein expression in both A549 and H1975 cells, and p-STAT3, PD-L1 protein expression could be rescued by STAT3 agonist, ML115 (**Figures 4A-C**).Besides, Flow cytometric analysis further demonstrated that treatment with SGC-0946 induced an downregulation of PD-L1 surface expression on lung cancer cells, an effect that was antagonized by co-treatment with ML115 (**Supplementary Figure S7**). confirming JAK1/STAT3 is the core molecular mechanism underlying DOT1L-induced PD-L1 up-regulation in LUAD. To exclude potential off-target effects of SGC0946, we then repeated our key mechanistic experiments using another DOT1L inhibitor, EPZ-5676 (27). Treatment with EPZ-5676 at a concentration of 4 nM for 48 hours significantly reduced H3K79me2 levels in both A549 and H1975 cells. Furthermore, time-course analysis of H3K79me2 protein levels at 4 nM across 12, 24, 48, and 72 hours revealed a gradual reduction over time until 48 hours, with no significant changes observed between 48 and 72 hours (**Supplementary Figure S9A**). Critically, EPZ-5676 treatment recapitulated the core phenotypes observed with SGC0946: downregulation of STAT3, phosphorylation of STAT3 at Tyr705, and reduced PD-L1 expression (**Supplementary Figure S9B**). The concordant results obtained with two chemically unrelated inhibitors (SGC0946 and EPZ-5676) indicate that the observed biological effects are specifically attributable to the inhibition of DOT1L methyltransferase activity.

To determine whether DOT1L inhibition improves the activity of immune cells, we focused on the potential regulatory impact of SGC0946 on PD-1+immune-cell subsets. Initially, we established co-culture systems incorporating A549 cells along with PBMCs, with the aim of mimicking a tumor-specific *in vitro* immune microenvironment. Further analysis of changes in the immune microenvironment revealed that SGC0946 intervention led to slightly increase in the levels of CD4+, CD8+ T cells in high dose-SGC0946 group (**Supplementary Figure S4**). Interestingly, the expression levels of CD3+PD-1+ T cells, CD4+PD-1+ T cells, and CD8+PD-1+ T cells were decreased in a SGC0946-does dependent manner (**Figure 4D**). These results collectively indicate that DOT1L fosters an immunosuppressive tumor microenvironment by potentiating STAT3-driven PD-L1 expression and T-cell exhaustion, while its inhibition may enhance antitumor immunity.

### 3.5 Pharmacological inhibition of DOT1L exerts antitumor effects in a LUAD tail vein lung metastasis model

**Figure 5:**
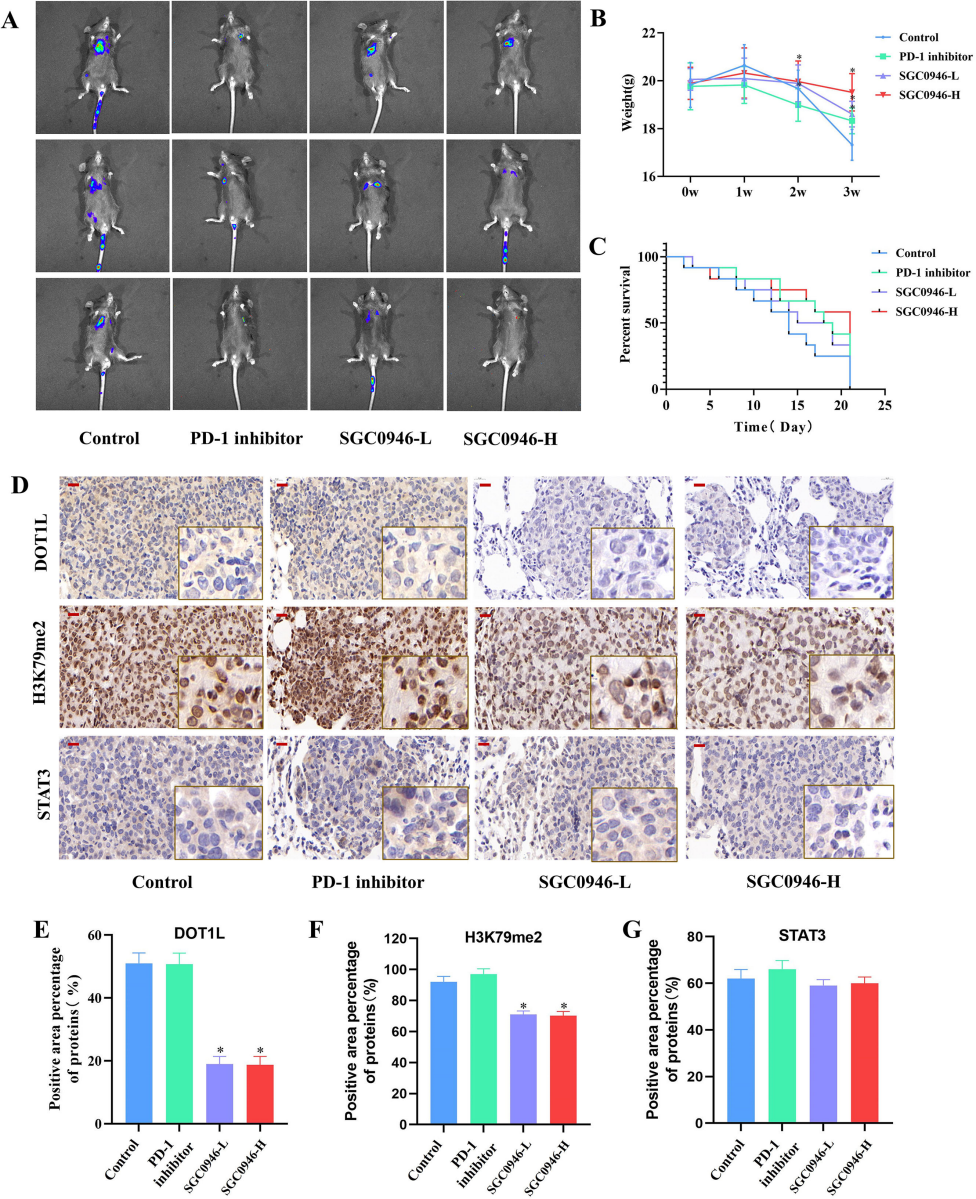
Pharmacological inhibition of DOT1L exhibits antitumor effects in a LUAD tail vein lung metastasis model. **(A)** Bioluminescence imaging shows *in vivo* evaluation of PD-1 inhibitor (200 μg i.p, twice weekly), SGC0946-L group(20 mg/kg), SGC0946-H group(40 mg/kg), in the tail-vein lung metastasis model. **(B)** Average weight of mice in in each group, control group(blue line), PD-1 inhibitor group(green line), SGC0946-L group(purple line), SGC0946-H group(Red line). **(C)** Survival estimates of mice in each group. **(D-G)** IHC staining of DOT1L, H3K79me2, STAT3 protein expression in tumor tissues in each group. Red scale bar: 20 µm. SGC0946-L, low does group, SGC0946-H, high does group. *P < 0.05.

Successful immune checkpoint blockade therapy was associated with increased anti-tumor immune cells. Since DOT1L transcriptional level is positively correlated with the canonical immune evasion effectors, suggesting its potential role as an epigenetic orchestrator of tumor immune escape, which underlies pharmacological inhibition of DOT1L may serve as a promising ICIs therapy. To evaluate translational implications, we employed tail vein lung metastasis model, and treated with pharmacological DOT1L inhibition (32) or PD-1 inhibitor (33). The PD-1 inhibitor group demonstrated the most potent *in vivo* antitumor efficacy, as evidenced by the lowest bioluminescence intensity in live imaging among all groups (**Figure 5A**). Both low- and high-dose SGC0946 treatments exhibited antitumor effects, with luminescence signals lower than those in the model control group, and the high-dose group showed superior efficacy compared to the low-dose group(**Supplementary Figure S5**). In terms of body weight, and mice in the high-dose group maintained significantly higher body weights (**Figure 5B**). Additionally, the high-dose SGC0946 group achieved the longest median survival (14 vs 18 vs 15 vs 21 day, control vs PD-1 inhibitor vs SGC0946-L vs SGC0946-H) (**Figure 5C**). These results suggest that while DOT1L inhibition (via SGC0946) may not exert as robust an antitumor effect as PD-1 blockade, its broader immunomodulatory functions likely contribute to a more beneficial systemic regulation, thereby improving overall survival and physiological conditions in mice.

We then performed IHC staining to assess the protein expression levels of DOT1L, H3K79me2, and STAT3 in tumor tissues. Our results demonstrated that SGC0946 significantly reduced the expression of DOT1L and its epigenetic marker H3K79me2 in both low and high does group. But the expression of STAT3 showed a modest reduction tendency in the SGC0946-treated group, however, there were no significant differences across the groups (*P* > 0.05) (**Figures 5D–G**). These findings further demonstrated that SGC0946 inhibited DOT1L and H3K79me2 expression *in vivo*, but the discrepancy in STAT3 expression between *in vitro* and *in vivo* experiments may be attributed to the intrinsic heterogeneity of the *in vivo* microenvironment.

### 3.6 Impact of DOT1L inhibition on the lung cancer immune microenvironment

**Figure 6:**
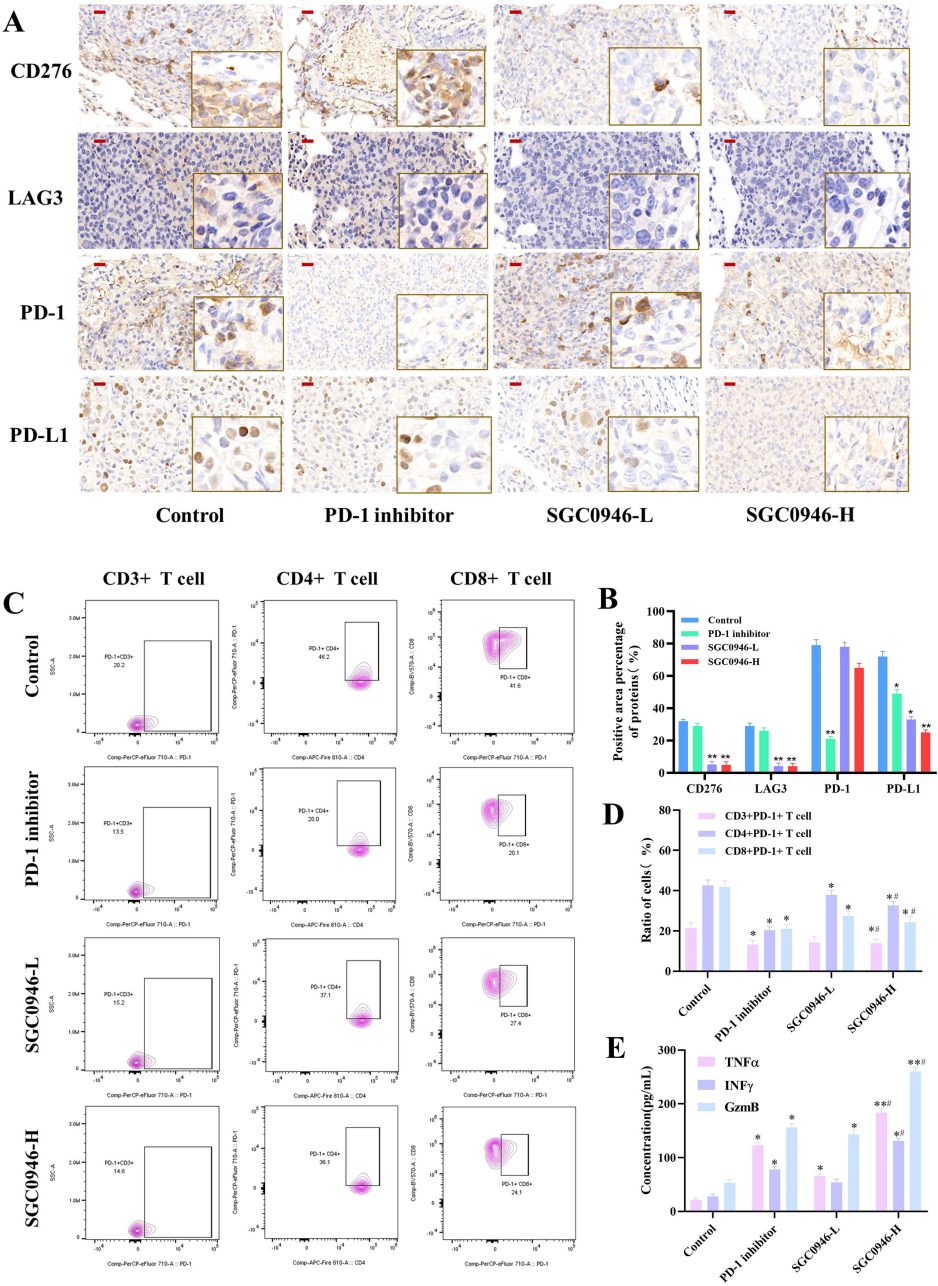
Impact of DOT1L Inhibition on the Lung Cancer Immune Microenvironment. **(A, B)** IHC staining of CD276, LAG3, PD-1, PD-L1 protein expression in tumor tissues. **(C, D)** Flow cytometry detected PD-1^+^ T-cell populations in tumor tissues. **(E)** ELISA assays detected the levels of cytotoxic cytokines (TNF-α, IFN-γ, granzyme B) in tumor tissues. Red scale bar: 20 µm. SGC0946-L, low does group, SGC0946-H, high does group. *P < 0.05, **P < 0.001 versus control. #*P* < 0.05 versus PD-1 inhibitor.

To further elucidate the immunomodulatory effects of DOTL1 inhibition, we focused on its influence on the tumor immune microenvironment. IHC analysis demonstrated that pharmacological DOT1L inhibition by SGC0946 reduced the expression of CD276, LAG3 significantly in both low and high does group, and reduction of PD-L1 was observed significantly in high does group (**Figures 6A, B**). Suggesting DOT1L plays a broader regulatory role in immune evasion.

Further characterization by flow cytometry revealed that SGC0946 treatment slightly enhanced CD8+ T-cell infiltration (**Supplementary Figure S6**), indicating a potential reinvigoration of antitumor immunity. Notably, both SGC0946 and PD-1 inhibitor significantly reduced the PD-1+ T-cell subset, with the PD-1 inhibitor exerting a more pronounced suppressive effect (**Figures 6C, D**).

Additionally, ELISA assays showed that both SGC0946 and PD-1 inhibitor elevated the levels of cytotoxic cytokines and effector molecules (TNF-α, IFN-γ, and granzyme B), with the SGC0946-H group induced a more pronounced increase (**Figure 6E**). These findings suggest that DOT1L inhibition remodels immune checkpoint expression significantly *in vivo*, supporting a potential epigenetic and immunotherapeutic approach for LUAD.

### 3.7 Clinical correlation of DOT1L expression with immune checkpoints in LUAD

**Figure 7:**
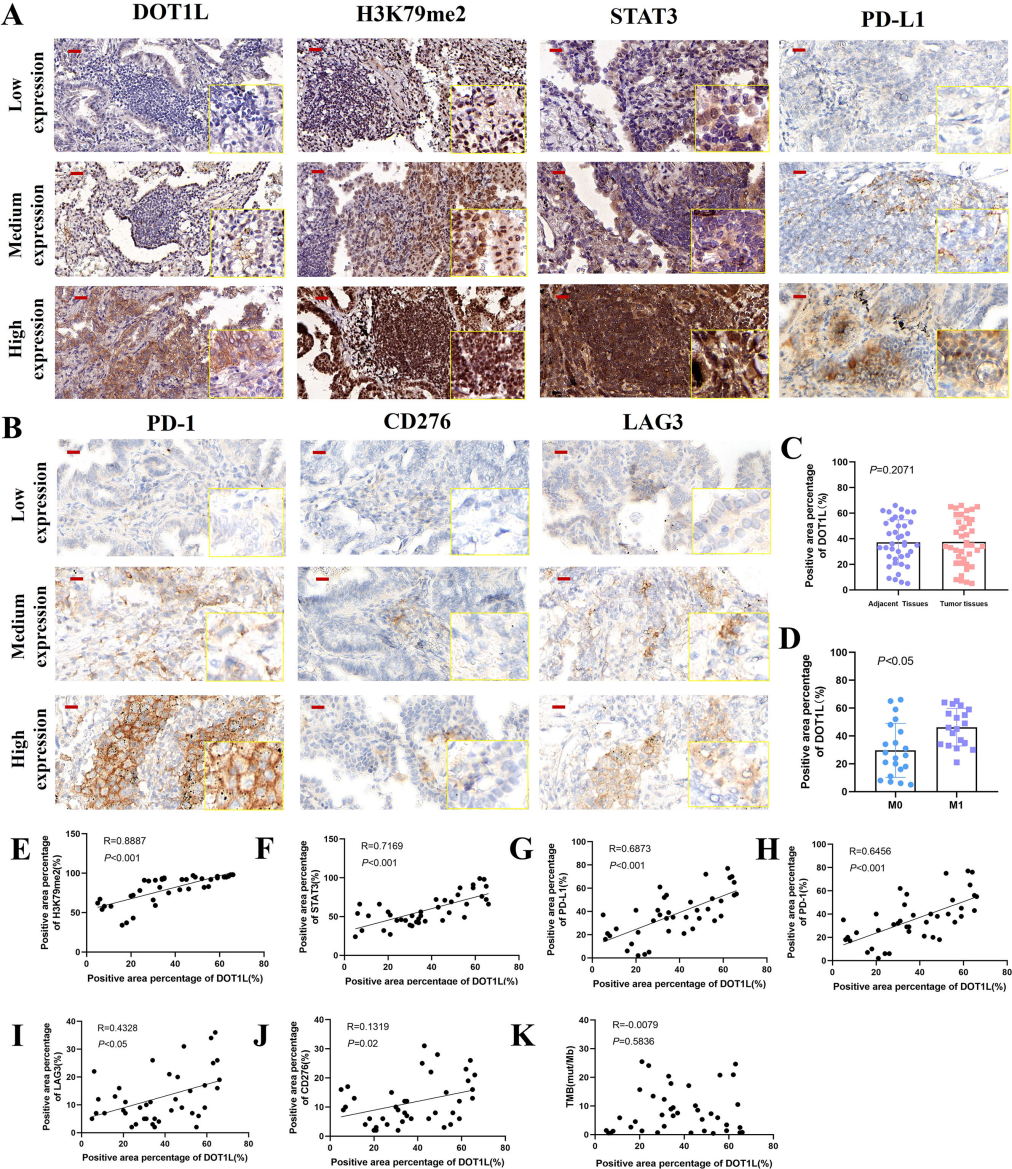
Clinical association of DOT1L expression with metastatic progression and immune checkpoint regulation in LUAD. **(A, B)** IHC analysis of DOT1L and associated markers (H3K79me2, STAT3, PD-L1, PD-1, CD276, and LAG3) in LUAD tissues stratified by DOT1L expression levels. **(C)** Comparison of DOT1L expression between tumor and adjacent normal tissues. **(D)** DOT1L expression in stage IV metastatic LUAD compared to non-metastatic cases. **(E-J)** Pearson correlation analysis between DOT1L and immune checkpoint/epigenetic markers. **(K)** Pearson correlation analysis between DOT1L expression and TMB.

Postoperative and puncture specimens from 40 cases of LUAD were collected, and the protein expression levels of DOT1L, H3K79me2, STAT3, PD-L1, PD-1, CD276, and LAG3 were evaluated via IHC. The results showed that DOT1L expression was comparable between tumor and adjacent normal lung tissues (**Figure 7C**). However, in stage IV LUAD with distant metastasis, DOT1L expression was higher than in cases without metastasis (**Figure 7D**). Based on DOT1L expression levels, the cases were stratified into low-, medium-, and high-expression groups. Notably, H3K79me2, STAT3, PD-L1, and PD-1 exhibited a progressive increase in expression alongside elevated DOT1L levels. In contrast, LAG3 showed no significant difference between medium- and high-expression groups but was markedly downregulated in the low-expression group. CD276 expression remained relatively stable across all groups (**Figures 7A, B**). Pearson’s correlation analysis showed that H3K79me2, STAT3, PD-L1, PD-1 expression was significantly correlated with expression of DOT1L (**Figures 7E-H**), LAG3 expression was positively correlated with expression of DOT1L (**Figure 7I**). But CD276 expression showed no significant correlation with expression of DOT1L (**Figure 7J**). Besides, high TMB (Tumor Mutational Burden) is often associated with increased neoantigen production, which may enhance response to ICIs (34), we didn’t find the correlation between DOT1L and TMB (**Figure 7K**). These findings suggest that DOT1L may play a role in promoting tumor progression in an epigenetically dependent way, particularly in advanced metastatic LUAD.

## 4 Discussion

This study elucidates the multifaceted role of DOT1L in LUAD, particularly in immune evasion and tumor progression. Our findings suggest that DOT1L, through its histone methyltransferase activity, epigenetically modulates key immune-related genes, fostering an immunosuppressive TME and contributing to poor clinical outcomes in LUAD patients. First, we observed that DOT1L expression tends to be higher in metastatic LUAD samples and is significantly associated with worse overall survival, indicating its potential role in tumor aggressiveness. Although DOT1L did not correlate with tumor stage or differentiation grade, but single-cell RNA-seq analyses revealed that its impact on immune regulation was evident, further supporting its tumor-promoting function.

Mechanistically, ChIP-seq demonstrated that DOT1L-mediated H3K79me2 modifications are enriched at promoters of immune evasion-related genes, including JAK1, STAT3, RELA, LAG3, and CD276. These genes are crucial in PD-L1/PD-1 checkpoint signaling, T-cell exhaustion, and cytokine-mediated immunosuppression. Pharmacological inhibition of DOT1L with SGC0946 reduced H3K79me2 levels and downregulated STAT3, JAK1, and RELA, confirming DOT1L’s role in sustaining an immunosuppressive TME via the JAK1/STAT3 axis. Notably, DOT1L inhibition also decreased PD-L1+ LUAD cell and PD-1+ T-cell populations and enhanced cytotoxic cytokine secretion (TNF-α, IFN-γ, granzyme B), suggesting partial restoration of antitumor immunity. Since STAT3 as a transcriptional activator of PD-L1 in lung cancer (31), our findings introduce DOT1L-mediated H3K79me2 directly enriches at the STAT3 promoter, elevating its transcription and thereby amplifying the downstream PD-L1 expression program. This positions DOT1L as another modulator within the known STAT3-PD-L1 axis.

*In vivo*, SGC0946 exhibited significant antitumor effects in a LUAD metastasis model, improving survival and reducing immune checkpoint expression (PD-L1, LAG3, CD276). While PD-1 blockade showed stronger tumor suppression, DOT1L inhibition provided broader immunomodulation, potentially offering a complementary strategy to ICIs. Clinical validation in LUAD specimens further confirmed that DOT1L expression correlates with H3K79me2, STAT3, PD-L1, and PD-1, particularly in metastatic cases, reinforcing its relevance in advanced disease.

However, limitations exist. Based on the above data, we demonstrated that DOT1L primarily drives immune evasion in tumor cells through activation of the STAT3-PD-L1 axis. However, our study also observed correlations between DOT1L expression and several immune checkpoint molecules (e.g., LAG3, PD-1), these correlations likely reflect differences in immune cell infiltration within the tumor microenvironment (35, 36), rather than direct transcriptional regulation of these genes by DOT1L in cancer cells. Besides, the lack of stable DOT1L-knockdown models hindered mechanistic validation, and the *in vivo* heterogeneity may explain discrepancies in STAT3 modulation. Additionally, for the immune co-culture experiments, we employed the A549 cell line which exhibit relatively lower baseline PD-L1 expression compared to some other cells (e.g., H1975) (37), the magnitude of PD-L1–dependent effects may be more pronounced in LUAD cells with higher endogenous expression. While DOT1L inhibition reshaped the TME, its standalone efficacy was modest compared to PD-1 blockade, suggesting combinatorial approaches may be necessary. These observations provide new clues and directions for future research into the potential role of DOT1L in modulating immune cell function, and DOT1L’s interaction with other epigenetic regulators and its potential synergy with ICIs.

In conclusion, our study highlights DOT1L as an epigenetic driver of immune evasion in LUAD, acting through H3K79me2-mediated activation of immunosuppressive pathways. Targeting DOT1L may represent a novel therapeutic strategy to overcome immune resistance and improve outcomes in LUAD, particularly in metastatic settings. Further preclinical and clinical investigations are warranted to optimize DOT1L-directed therapies in lung cancer immunotherapy.

## Data Availability

The data presented in the study are deposited in the NGDC (National Genomics Data Center) GSA (Genome Sequence Archive) database, and the relevant accession number is subHRA025977.
