# Supplementary material for: DOT1L promotes immune evasion in lung adenocarcinoma through H3K79me2-mediated epigenetic activation of immune checkpoints
Source: Front Immunol. 2026 Feb 26;17:1719299. doi: 10.3389/fimmu.2026.1719299 (PMC12979096; doi:10.3389/fimmu.2026.1719299)
Supplement: Supplementary file 1 [file DataSheet1.docx]

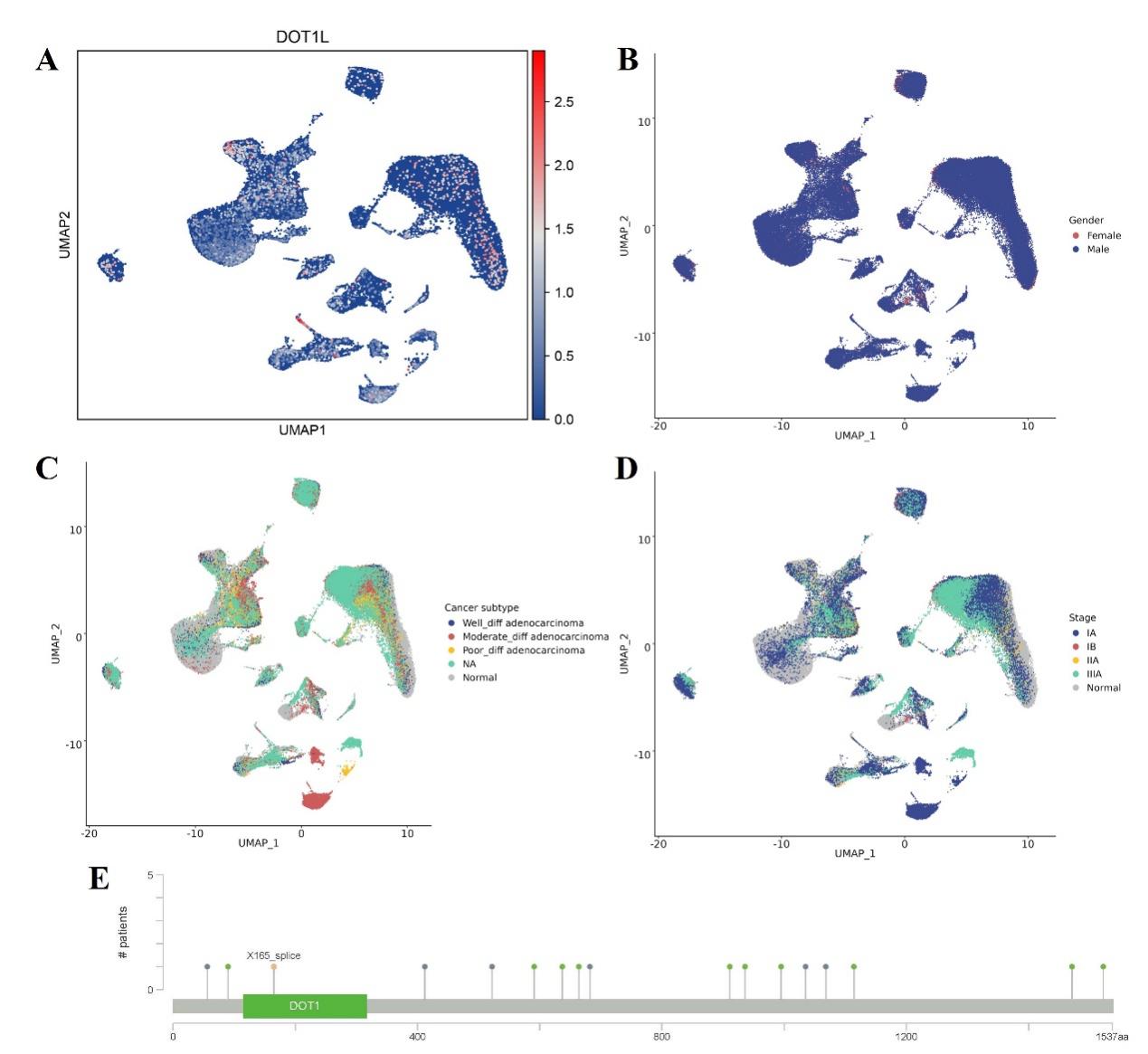


Figure S1. Association between DOT1L expression and clinicopathological features in LUAD. (A-B) Single-cell expression profiling exhibits DOT1L expression among male and female in LUAD. (C) Single-cell expression profiling exhibits DOT1L expression among well, moderate, poor differentiation LUAD and normal lung tissues. (D) Single-cell expression profiling exhibits DOT1L expression in stage Ⅰ, Ⅱ, Ⅲ, Ⅳ in LUAD.

(E) Analysis of mutation frequency and mutation site of DOT1L in LUAD cases.


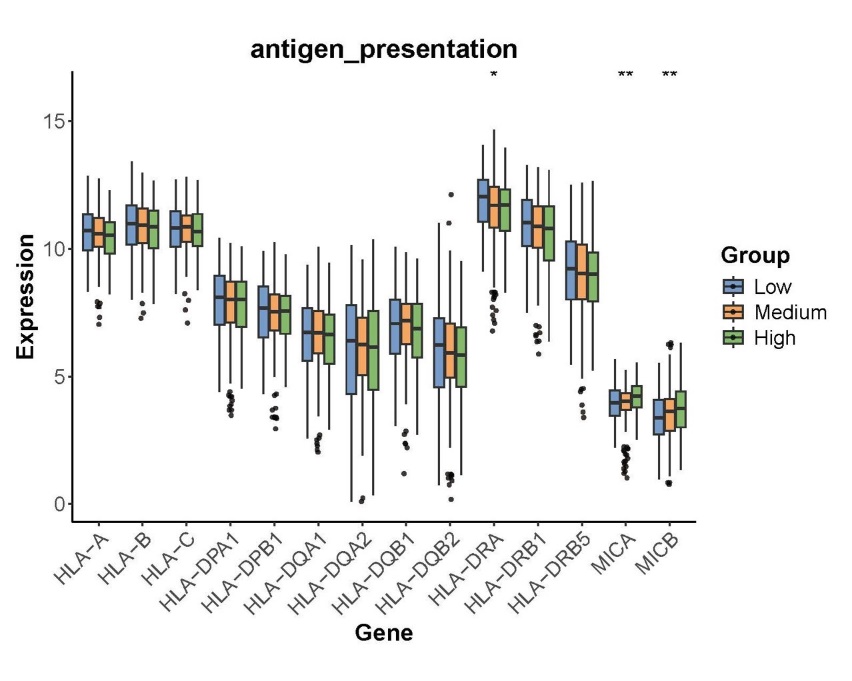


Figure S2. Evaluation of DOT1L Expression with antigen_presentation in LUAD by ssGSEA(**P*<0.05, ***P*<0.01).


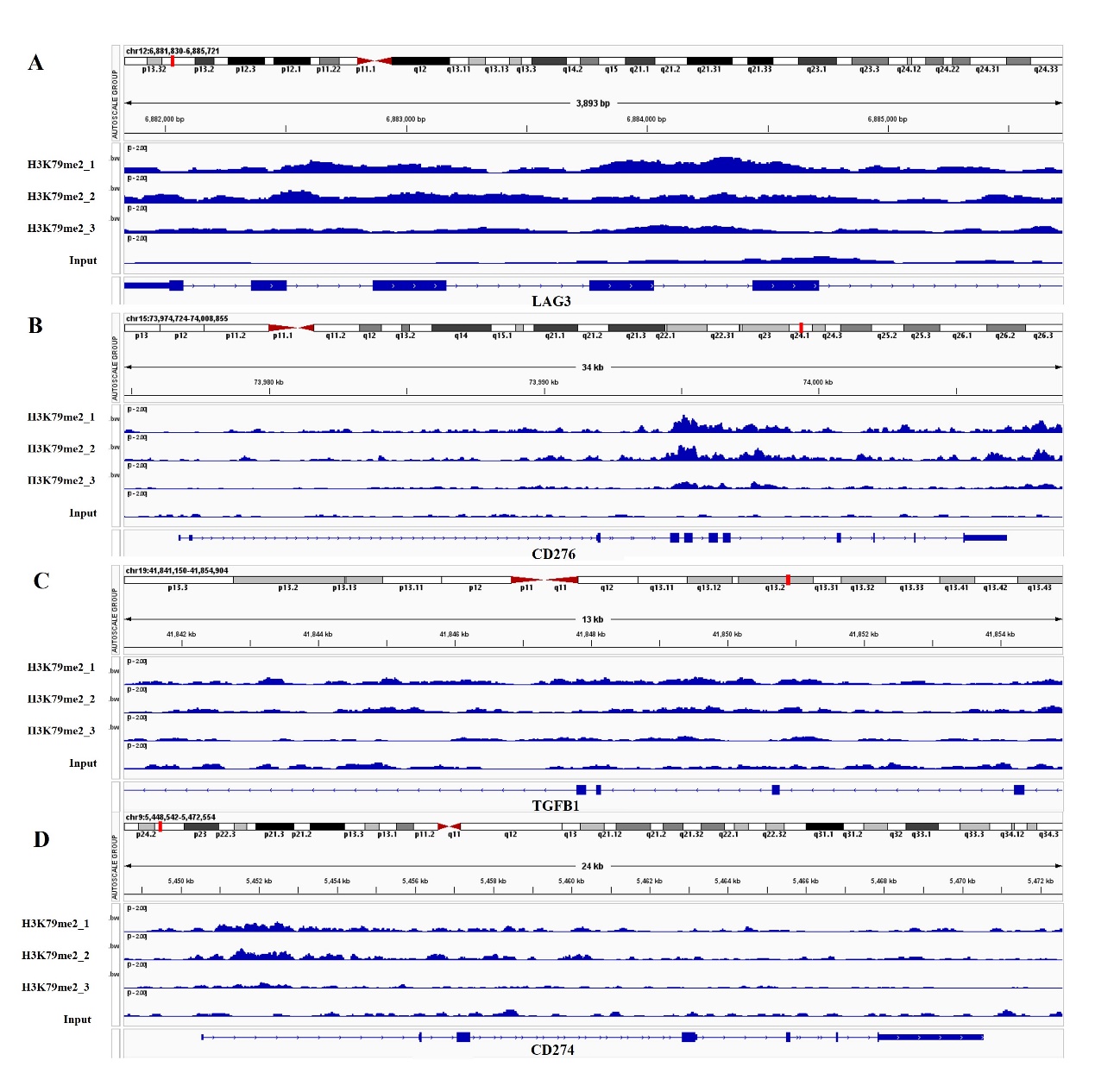


Figure S3. ChIP-seq profiles showing H3K79me2 enrichment in the gene regions of LAG3, CD276, TGFB1, and CD274 in A549 cells.


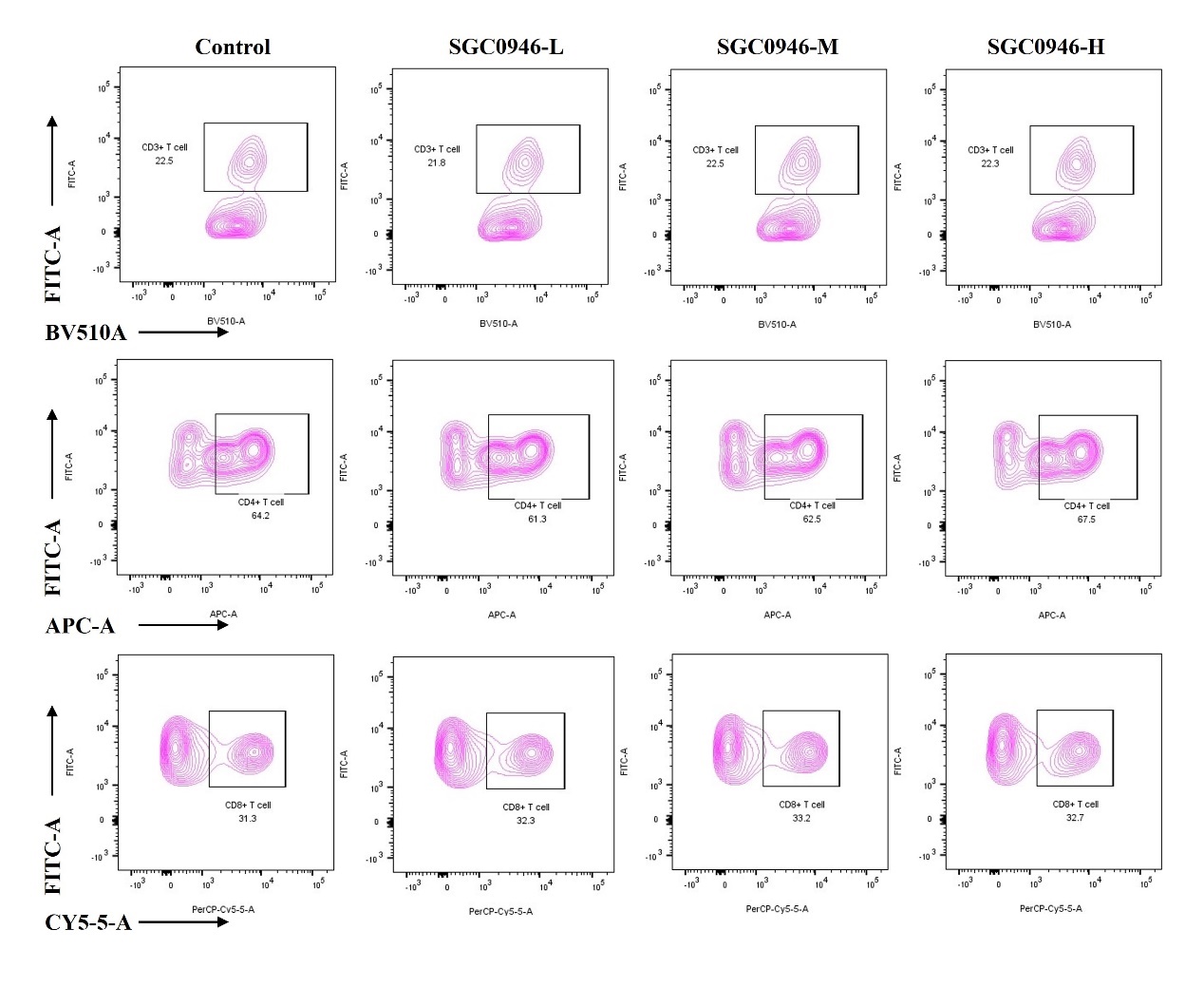


Figure S4. Flow cytometry analysis of T cell subsets in the co-cultured system of PBMCs with A549 cells


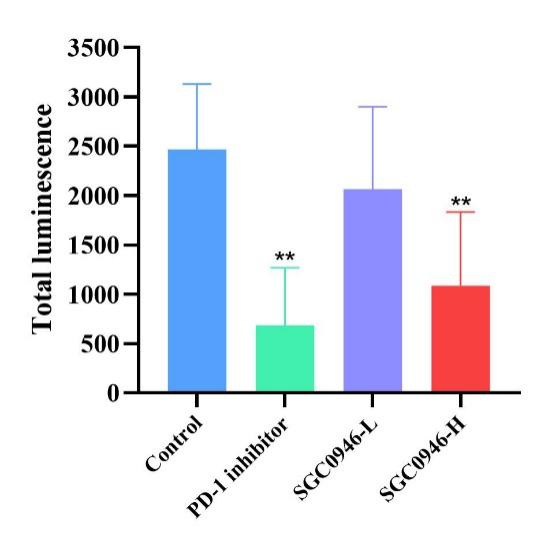


Figure S5. Luminescene of mice in in each group. ***P* < 0.001.


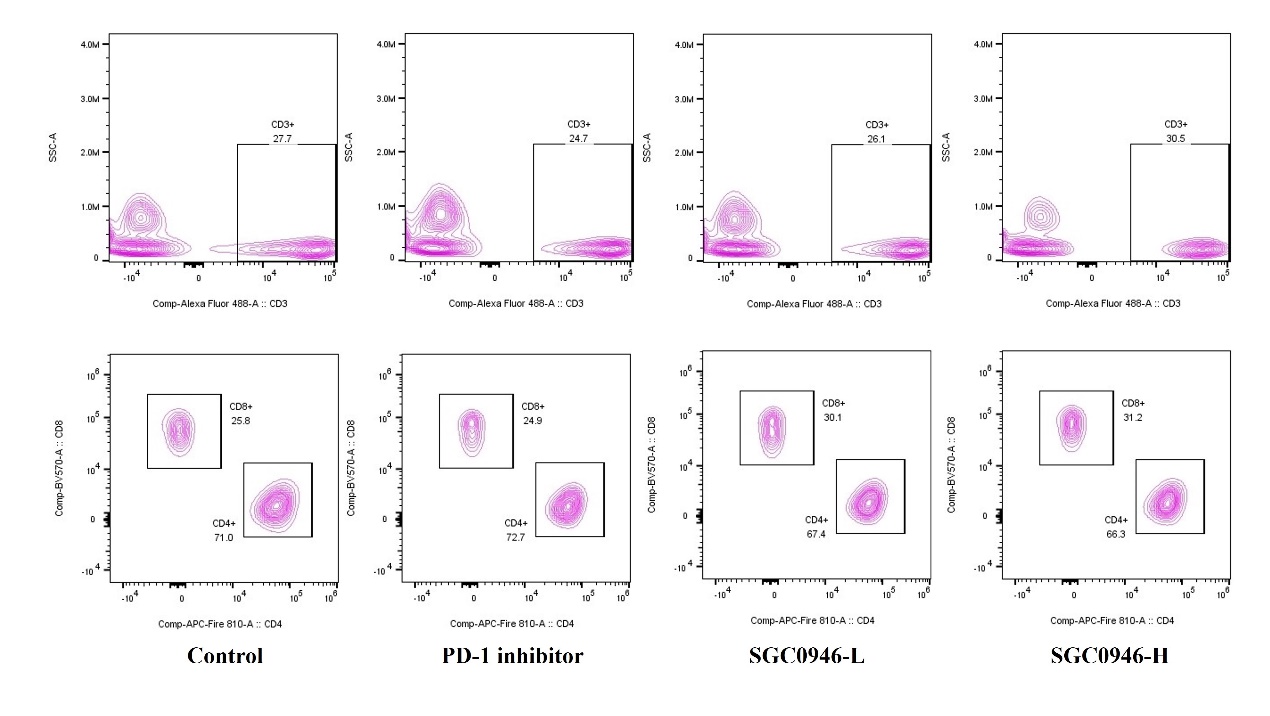
Figure S6. Flow cytometry detected T-cell populations in the tumor tissues in each group.


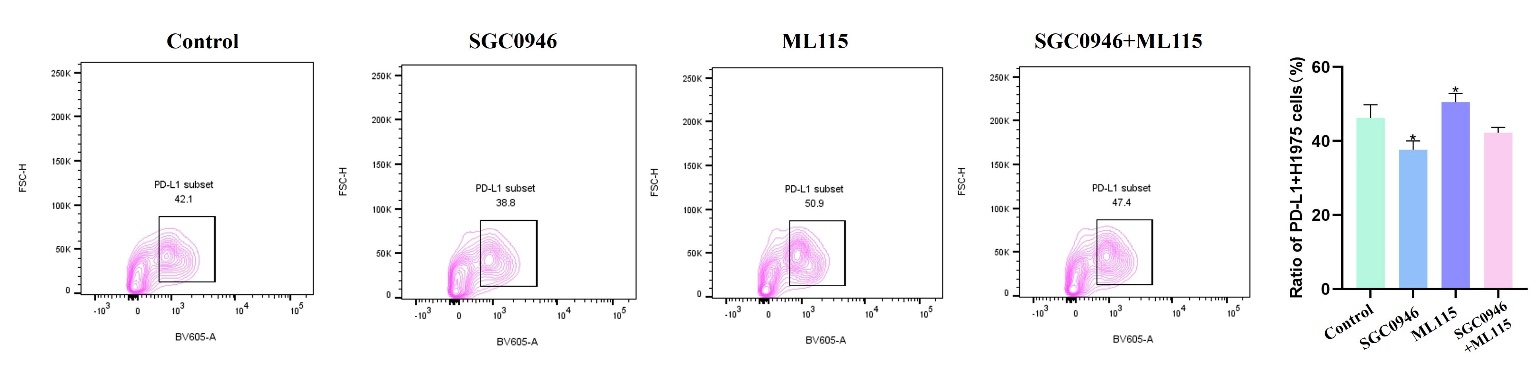
Figure S7. Flow cytometry detected PD-L1^+^H1975 cell populations in each group.


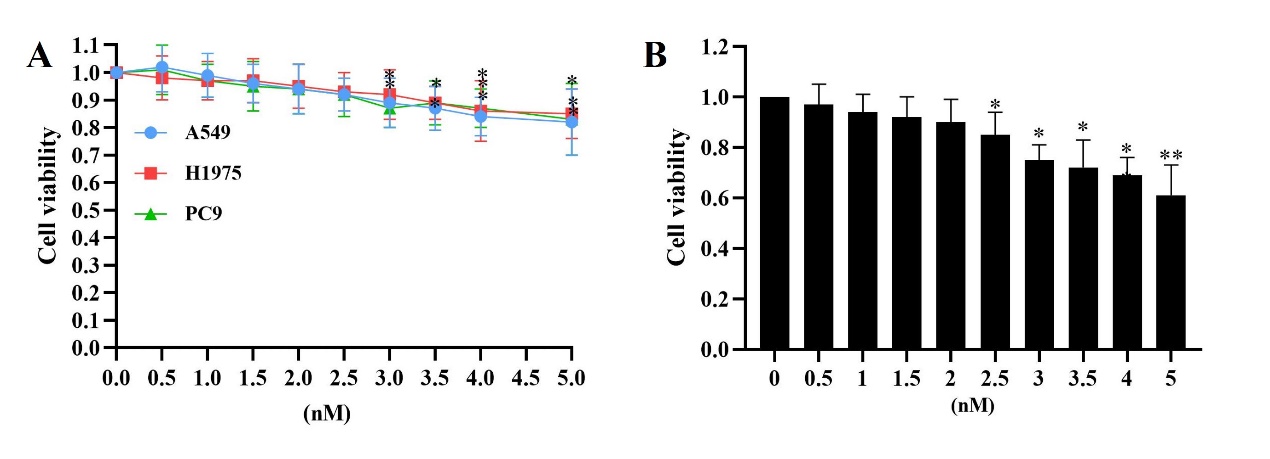
Figure S8. Viability of LUAD cells and PBMCs following treatment with SGC0946.


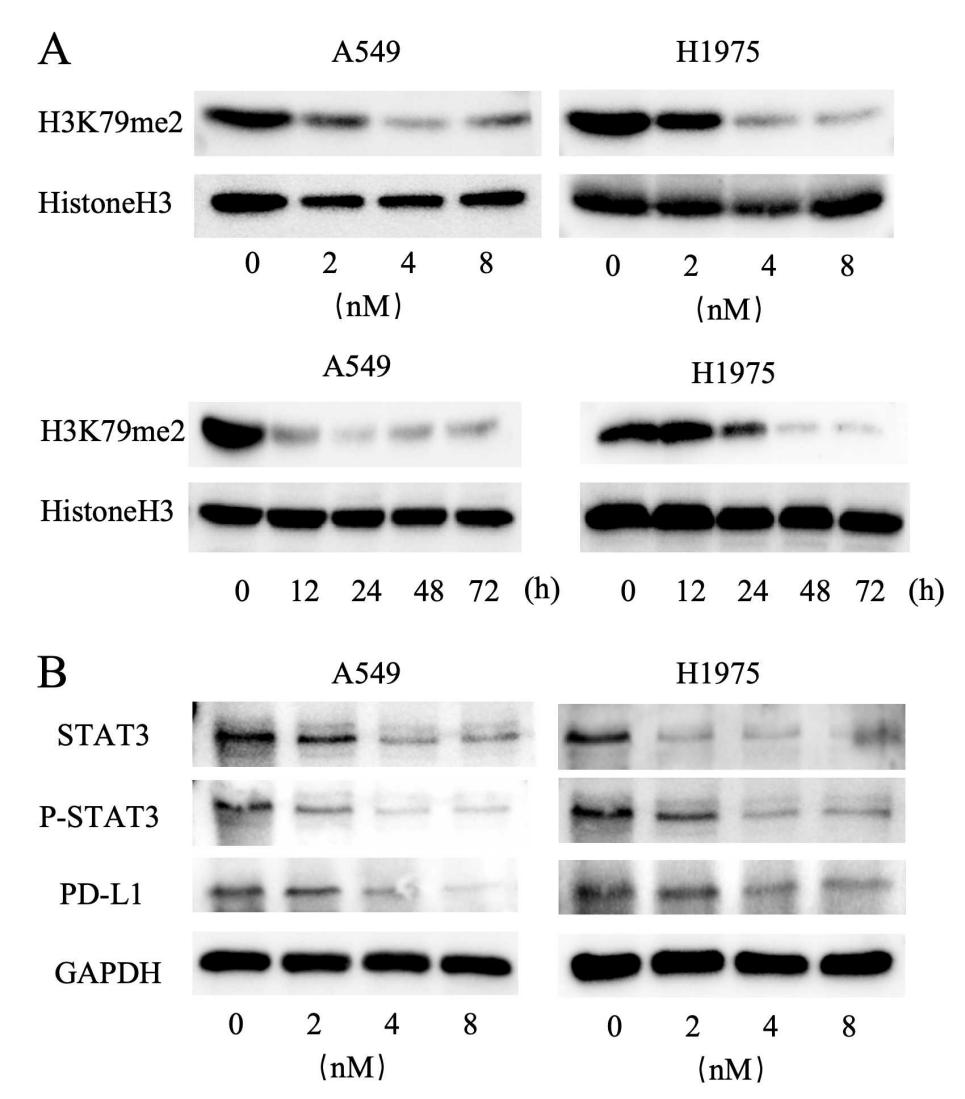


Figure S9. Western blot analysis detected (A-B)H3K79me2 protein levels after treatment with the DOT1L inhibitor EPZ-5676. HistoneH3 served as a loading control. (B) STAT3, p-STAT3, PD-L1 protein levels after treatment with EPZ-5676. GAPDH served as a loading control.


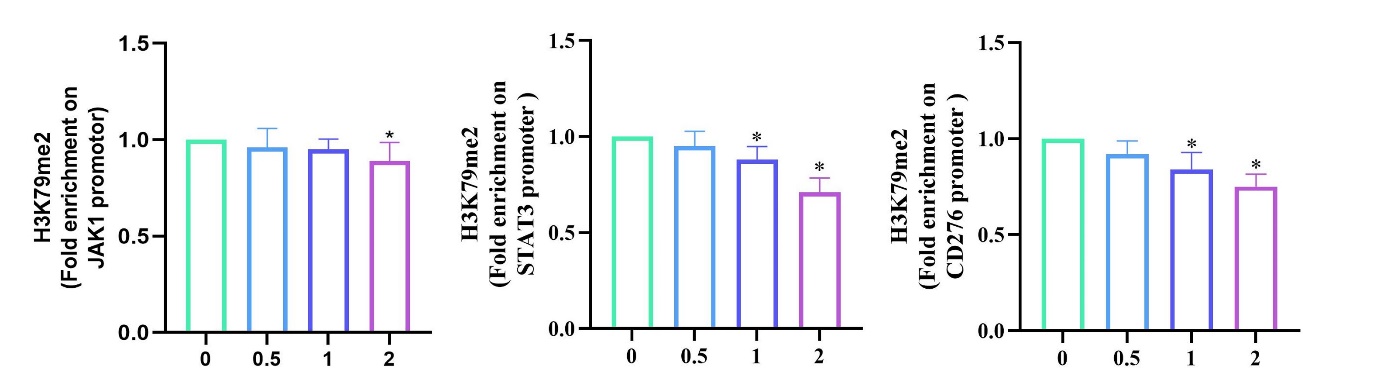


Figure S10. ChIP-qPCR analysis of H3K79me2 enrichment at the promoter regions of JAK1, STAT3 and CD276.
